# Clinic of Professor Dunglison

**Published:** 1845-06

**Authors:** Samuel G. White

**Affiliations:** Georgia


### CLINICAL LECTURES AND REPORTS.

### PHILADELPHIA HOSPITAL.

*Saturday, January* 25, 1845.

### CLINIC OF PROFESSOR DUNGLISON.

Reported by Dr. Samuel G. White, of Georgia.

Professor Dunglison commenced his lecture with some observations on

DISEASES OF THE NERVOUS SYSTEM, its physiology having been considered by him during the week in the Jefferson Medical College.

The first case, to which the attention of the class was directed, was that of a female suffering from the effects of

RAMOLLISSEMENT OF THE BRAIN AND CONSEQUENT HEMORRHAGE, who has been in the Hospital for a great length of time, and was seen by the class of last session on several occasions.

This disease—softening of the encephalic neurine—has only been investigated within the present century, prior to which it seems to have been unknown. About the year 1820, and subsequently, several treatises appeared, since which the subject has continued to occupy the attention of medical observers.

The diagnosis of this affection is by no means always easy, though it is often characterised by marked phenomena. In its commencement there is generally an intense fixed local pain, various in its seat, but mostly frontal, which does not yield to ordinary treatment. This continues for a length of time and may then disappear. When the softening has occurred, there is more or less disturbance of the intellectual faculties, with loss or depravation of sensation and motion. Sometimes these disturbances exist in a marked degree, and constitute the main expressions of the disease.

We can readily comprehend how sensation and motion, and the intellectual and moral manifestations, may be influenced by disease situate in the cerebral hemispheres—which are most commonly involved in ramollissement of the neurine—when it is remembered that the sensory and motor tracts of the spinal column are prolonged upwards to the periphery of the brain, and that the cerebrum is the seat of the mental faculties. The loss of sensation and motion may be incomplete or complete, according to the seat and extent of the injury. Sometimes sensation is morbidly increased, constituting *hyperæsthesia*, and, at others, totally destroyed, constituting *anæsthesia.* The degree to which the mental faculties are disturbed is also various. Every intellectual or moral act may be suspended, or the individual may be able to commence a sentence, but not to complete it. There is very often, indeed, incapability of concentrating the mind on any subject.

A recent writer—Durand Fardel—affirms, that the convolutions of the cerebrum are, in the majority of instances, the seat of softening, but it often implicates other portions of the encephalon.

From the decussation of the fibres of the medulla spinalis at the medulla oblongata, in their course to the brain, there is generally a cross effect produced—the side opposite that on which the disease is located being affected with loss of power. If, however, the disease attacks parts below the point at which this decussation takes place, the same side may be paralysed.

The professor here alluded to a case on which he had been recently consulted, and which, from the mental disturbance, the depravation of the senses and of motion, he had no hesitation in pronouncing it to be one of softening of the cerebral neurine. Whenever, indeed, the symptoms already mentioned exist, without any other cause to which they may be referable, we may be justified in the presumption, that they are expressions of the disease under consideration.

Should this softened condition of the brain remain for any length of time—which is usually the case—a transudation of blood is apt to occur, owing to the diminished support afforded to the vessels by the softened encephalic tissue. Wherever blood vessels pass through parts of little consistency, and are therefore feebly supported, hyperæmia and consequent hemorrhage is apt to supervene This is the case with the vessels of the schneiderian membrane of the nose, and it accounts for the frequent occurrence of epistaxis in plethoric persons, after slight exercise, as well as in those who, from malarious or other influence, are liable to irregularities in the circulatory movements. Should this transudation take place in the substance of the brain, we have all the phenomena of hemiplegia, which is thus a common result of encephalic softening.

In the patient—presented by the lecturer before the class—after the symptoms of softening had persisted for some time, great deviation of the eyes occurred; and the class would observe that she is at present affected with considerable strabismus. This, the professor thought, is dependent on the disease implicating the optic lobes or tubercula quadrigemina. Formerly, it would have been referred exclusively to the thalami nervorum opticorum, under the view once entertained that those bodies are the origin of the nerves of sight. This condition of the eyes was followed by paralysis of one side of the body, whence it was inferred, that encephalic hemorrhage had taken place, although it might have been owing to extension of the softening. Occurring suddenly, however, the paralysis was more probably dependent upon sanguineous transudation. She has had several attacks of this kind, doubtless arising from the weakened condition of the blood vessels, and want of support, in the manner already mentioned. At present there is complete paralysis of the right arm and leg, but the sensation remains intact. The incapability of transmitting, through volition, the locomotive influx to the muscles, is complete. This, consequently, is a case of disease affecting the motor nerves alone, the sensory remaining undisturbed. Her voice is greatly altered, and a deviation of the axis of the eyes, which, as before remarked, still exists, was previously much more considerable. In this, as in almost all cases of a similar character, there is torpor of those functions that are executed not under the immediate influence of the brain, but rather of the spinal column. The organic functions, in general, are not imperfectly performed; but there is obstinate constipation, which is but little influenced by any remedy. This, indeed, constitutes a great difficulty in the treatment.

Another phenomenon of interest is a great increase in the salivary secretion, whenever the constipation is most marked. It collects in the mouth, and, owing to the torpor of the different nerves, it is not swallowed as it accumulates, but flows out of the mouth in considerable quantity.

As to the precise pathology of ramollissement of the brain, there is discrepancy of opinion. It is referred by some to inflammation of the neurine; but the lecturer thinks this is not always present, and, when it is, it is probably almost always chronic. He thinks that, independently of inflammatory action, there may be an alteration in the nutrition of the part, which may modify the consistence in such a manner as to give rise to softening.

The treatment of the affection is by no means satisfactory. In the vast majority of instances nothing can be done, and the individual goes on from bad to worse, until death terminates the scene. Still, cases do recover, and in every case we are called upon to prescribe according to the indications. Should symptoms of inflammation or hyperæmia of the brain exist, cups may be applied to the back of the neck; or other revellents, as blisters, may be used in the same situation.

But manifestly these can be of little, if any, benefit when the disease has proceeded to the disorganization of the neurine. In such case we are compelled to treat the symptoms as they arise. If any good is to be accomplished, it must be by a modification of nutrition, which time and the instinctive actions can alone accomplish. The prognosis is in all cases unfavourable,—the patient rarely recovering. The female, whose case is under consideration, the lecturer thinks, will gradually — perhaps suddenly—proceed from worse to worse, and finally die. It is strange, indeed, that she has survived so long.

The next patient brought before the class was affected with an analogous lesion, and was introduced to exhibit the marked effects of CEREBRAL HEMORRHAGE. It was that of a female, almost 30 years of age, labouring under

### HEMIPLEGIA.

Generally, hemiplegia is observed in the adult or aged, but sometimes young persons are attacked with it. It seems to be influenced by atmospheric temperature, as it is more common in climates where the temperature is elevated. In British India, young Europeans are not unfrequently destroyed by it, or compelled to return to their native country, to drag on a burthensome existence. Yet in other countries, where the temperature is equally high, it is not as often seen, and hence there seems to be something in the locality also that predisposes to it, but what this is has not been, as yet, ascertained.

Hemiplegia is generally employed to denote the paralysis resulting from cerebral hemorrhage. Apoplexy, also, is often used in the same sense as cerebral hemorrhage, but this is objectionable, inasmuch as the aggregate of symptoms denoting apoplexy may be produced by hyperæmia, or by pressure of any kind on the encephalon. The term apoplexy, indeed, as usually employed, denotes an aggregate of functional phenomena, not any special lesion. Of late years, however, it has been more employed in the sense of interstitial hemorrhage, and hence we have apoplexy of the brain, apoplexy of the lungs, &c. &c.

The professor here alluded to the practice, too prevalent—and yet at times unavoidable—of applying names to symptoms rather than to the diseased condition. This is the case, as just stated, in regard to apoplexy. It is so termed because the patient falls down. But, manifestly, this falling down—as just shown—may be dependent on a variety of causes. The same may be said of the terms indigestion and constipation, so often employed. They might, with great propriety, be abolished altogether, as they constitute nothing more than expressions of disease; simple statements of the fact, that the individual has impaired digestion or confined bowels. They may be expressions of the most opposite organic or functional conditions. Again, it is a great error to dwell on effects, to the neglect of causes. In every instance, where this is practicable, the pathological state that gives rise to any phenomenon should be determined, as on a correct knowledge of this can proper treatment alone be based. At times, however, there is great difficulty in discovering the pathology; and the disease is named from the most characteristic or prominent symptom or symptoms. This is especially the case with the numerous class of the neuroses—and hence we have the terms palsy, tremors, tetanus, hydrophobia, hysteria, &c., which could not be abolished without great confusion resulting. In our ignorance, indeed, of the pathological aberration they are indispensable. The lecturer referred, in terms of commendation, to some recent remarks on "Diseases and Symptoms," contained in the Saint Louis Medical and Surgical Journal for December 1, 1844, by a former intelligent and zealous resident physician of this Institution—Dr. McPheeters—now practising his profession in Saint Louis, and one of the Editors of the Journal referred to.

In most cases of hemiplegia, there is the cross effect before alluded to. It would not appear, that the whole of the fibres of the medulla spinalis decussate, but the majority of them certainly do. Hence we can understand that, in some rare cases, there may be paralysis of the same side, owing, no doubt, to the lesion implicating only the non-decussating fibres.

The seat of cerebral hemorrhage is very various; but generally in, or on a level with, the corpora striata, or thalami nervorum opticorum. Sometimes, it occurs on one side of the medulla oblongata, and the professor narrated a case, in which he had been recently consulted, where he believed this to be its seat. The patient, whilst walking in the street, discovered that he had some difficulty in directing his motions properly, and was so vertiginous, that he was compelled to stop and support himself on a projection in the street. He recovered so as to proceed home; but from the first almost entirely lost the sense of hearing of one ear. The next day he was attacked with violent disturbance of the stomach, which passed off; but the loss of hearing continues, although not to the same extent. The lecturer gave it as his opinion, that some lesion had occurred on one side of the medulla oblongata, near the roots of the pneumogastric and auditory nerves. In this case, there would necessarily be no cross effect.

When the cerebellum is the seat of the hemorrhage, the paralysis is usually on the side opposite to the lesion; but it also may be on the same side.

In the case of the female before the class, the attack was preceded, as usual, by giddiness, which was soon followed by entire loss of motion over the extremities of one side. There was no apoplectic seizure. She has much improved, but there is still great difficulty in locomotion. There was also loss of sensation in the affected parts, which has nearly disappeared. She has had one or two returns of the cerebral hemorrhage—"paralytic strokes," as they are usually termed—though none very recently.

There is one point connected with the therapeutics of such cases, which the lecturer particularly desired the class to remember. He remarked, that it was customary—whenever an individual suddenly falls down deprived of sensation—for the bystanders to call for the lancet; and too frequently, in such cases, the physician yields to their solicitations, to the detriment of his patient. In many hemiplegic cases it is obvious that mischief may result from this course. When hemorrhage first occurs, the effect on the constitution is similar to that of concussion; a "shock" is communicated to the brain, and the person is in a state of depression, rather than of excitement. After this has passed off, symptoms of an opposite condition, or of compression, present themselves. In the stage of concussion, venesection can scarcely fail to prove injurious, and may be fatal. The indication is to establish reaction, which may be accomplished by gentle stimuli, frictions, revellents, &c. As soon as this is effected, or the evidences of compression appear, we have to decide as to the degree to which blood letting may be carried with propriety. The object to be attained by this means is two-fold,—first, to diminish the amount of blood and the activity of the circulation, and thus to prevent further effusion; and secondly, to promote the removal, by absorption, of the fluid already effused. During the apoplectic seizure, should general bleeding be deemed necessary, it may be aided by topical blood-letting by cups to the nape of the neck or the temples, elevating the head, and making a revellent impression by the application of cold to the head, sinapisms to the extremities, turpentine enemata, &c. &c. When the apoplexy—so called—has passed away, and the paralysis remains, we have recourse to other means, which are, however, for the most part of limited agency. If the pathology, indeed, be borne in mind we may understand how little can be effected by any remedy. Blood is effused into the brain, and its neurine is compressed; the clot remains; but soon a serous membrane is formed around it, which secretes a fluid; the clot thus becomes softened and absorbed, leaving a cell—the "apoplectic cell"—which is at once the evidence of previous extravasation and subsequent absorption. What remedy can promote the removal of this clot, or cell? The professor remarked, that after long experience, he is compelled to say that, in the majority of cases, nothing essential can be done. The case must be left to the natural recuperative powers. There is no agent that can restore the lost nervous matter,—nothing the consequent loss of nervous power.

In confirmed cases of hemiplegia, strychnia, which is a tetanic or excitant of the nervous system, and especially perhaps of the excito-motory or reflex portion, has often been prescribed. But it should be remembered, that this potent article, by the stimulation it is capable of inducing, may do harm, and give occasion to a recurrence of the hemorrhage; and hence, in all cases of encephalic paralysis, great care should be had in its employment. In all local palsies and those dependent on lesions of the spinal marrow—as paraplegia—it is of more benefit, and the same dangers do not attend its administration. It may be used either internally, or endermically.

Besides the use of this article, recourse may be had to frictions, electricity, &c., to the paralysed limbs. But it need scarcely be said, that little or no good can result from exciting the sentient or terminal extremities of the nerves, for the removal of the encephalic affection. The seat of the lesion is manifestly beyond our reach. They are safe agents, however, and if no good results, certainly they can do no harm. Much injury may, however, be done by the employment of powerful counter-irritants, as Granville's lotion, &c. The sensibility of the surface is diminished, and its power of resisting morbid influences also lessened, so that vesication or gangrene more readily supervenes on the application of powerful local excitants.

In all these cases it is, perhaps, best to rely on the instinctive powers, treating only the most prominent symptoms as they arise. In process of time the effused fluid is absorbed, and more power is gained over the paralysed muscles. In too many cases, however, the muscles can never be effectively employed, and a fresh recurrence of the cerebral hemorrhage renders the individual still more helpless, or closes his unhappy existence.

The professor now alluded to a case of the affection described in a previous lecture, under the head of

JERKING RESPIRATION, which he then stated to be an important sign of tuberculosis. The subject of the present case, in which it was very marked, died, not, however, of the pulmonary disease, but of another affection, and tubercles were found to exist in considerable numbers, thus confirming the diagnosis. The specimen was accidentally thrown away during the unavoidable absence of the lecturer, from indisposition, and he had consequently no opportunity of exhibiting it to the class. He was indebted to Dr. Farquharson, one of the resident physicians to the wards, for the report of it. In addition to this condition of the lung, a tumour existed in the abdomen of the patient, immediately above the pubis, which, during life, was not manifested by any marked phenomena, although she died under symptoms of abdominal irritation. There was, towards the close, difficulty of respiration, but no acute suffering of any kind. The tumour, which occupied the inferior portion of the abdomen, was adherent to the parietes. The cavity of the peritoneum contained about a pint and a half of semi-purulent fluid, mingled with fæcal matter, which could also be expressed from an opening in the tumour. The large intestine was perforated in several places, the openings round, and of a dark red colour. From these the fæcal matter had manifestly escaped. Both the large and small intestines were adherent to the sides of the tumour, though distinct from it. On opening the tumour it was found to contain a hard nucleus about three inches in diameter, which was separated from the mass by soft tenacious matter, and a quantity of fæces. This nucleus consisted of a firm white homogeneous substance, composed principally of calcareous matter mixed with a soft mass. The uterus was somewhat enlarged, and a firm tumour, about three-quarters of an inch in diameter, was attached to its exterior, beneath the peritoneal reflection, moveable and fibrous in structure. The augmented volume of the organ was dependent on the existence of a fibrous tumour, having a broad base attached to one of the angles, and about the size of an egg. The lining membrane of the uterus was congested, but this was probably hypostatic.

The principal interest of this specimen consisted in the small amount of irritation excited by the presence of the diseased mass and fæcal effusion in the abdomen; and the case was strikingly confirmatory of the views inculcated at the last clinical lecture, in regard to the obscurity of many cases of chronic peritonitis.

The lecture was concluded by the exhibition of some interesting

PATHOLOGICAL SPECIMENS, from a female who died in the syphilitic wards.

Her death was rather sudden, and could be traced to no certain cause. It became, therefore, an important inquiry to determine it.

The lungs were found loaded with miliary tubercles, but there was no evidence during life of tuberculosis of the lung. Percussion—practised before the class—gave a duller sound than natural. It may seem strange, that the presence of so many tubercles should have excited so little irritation during life, yet we know that such is not unfrequently the case. The lecturer has seen the lungs most extensively studded with calcareous deposits, where not the slightest evidence had existed during life of any pulmonary lesion. It is certain that in the present case the condition of the lungs could not account for death. The heart presented nothing abnormous.

Before death, blood, the amount of which could not be estimated, was passed from the rectum, and a still greater quantity was discharged afterwards.

The cavity of the rectum communicated by ulcerated openings—probably syphilitic—with that of the vagina.

The lecturer remarked, that there seemed to be no assignable cause for death, unless the amount of hemorrhage was sufficient to produce it, which scarcely seemed probable. It is possible, indeed, that the fatal event might have occurred from syncope, and that in the diseased condition of the patient, the heart was unable to resume its action. At times, mercurial erethism induces a fatal result, but, although she was affected with syphilis, no mercury had been administered.

He referred to a case that fell under his cognizance, in which death occurred from the simple application of the unguentum hydrargyri oxidi rubri to eczema rubrum of the leg. The patient, whilst sitting up in bed, under mental emotion, became faint, fell back, and expired suddenly. The body was examined, and as no evident cause of death existed, it was presumed to have occurred from mercurial erethism. Many similar cases have been described by Mr. Pearson and others.

